# Influence of selenomethionine and omega-3 fatty acid on serum mineral profile and nutrient utilization of broiler chicken

**DOI:** 10.14202/vetworld.2015.164-169

**Published:** 2015-02-13

**Authors:** Pankaj Kumar, S. P. Tiwari, Tarini Sahu, Surendra Kumar Naik

**Affiliations:** Department of Animal Nutrition, College of Veterinary Science and Animal Husbandry, Anjora Chhattisgarh Kamdhenu Vishwavidyalaya, Durg, Chhattisgarh, India

**Keywords:** broiler chicken, nutrient utilization, omega-3 fatty acid, selenomethionine, serum mineral profile

## Abstract

**Aim:**

This study was conducted to investigate the effect of selenomethionine and omega-3 fatty acid on serum mineral profile and nutrient utilization of broiler chicken.

**Materials and Methods:**

The present study was a 2×3 factorial arrangement of two levels of selenomethionine (0 and 0.3 ppm) and three levels of omega-3 fatty acid (0, 0.5 and 1%). Day-old Vencobb broiler chicks (n=180), were randomly assigned in six treatment groups. The experiment lasted for 42 days. Treatment groups followed of: Group I was a control. Group II, III, IV, V and VI were supplemented with 0 ppm selenomethionine with 0.5% omega-3 fatty acid, 0 ppm selenomethionine with 1% omega-3 fatty acid, 0.3 ppm selenomethionine with 0% omega-3 fatty acid, 0.3 ppm selenomethionine with 0.5% omega-3 fatty acid and 0.3 ppm selenomethionine with 1% omega-3 fatty acid, respectively. Linseed oil was used as a source of omega-3 fatty acid while sel-plex is used for selenomethionine supplementation.

**Results:**

Significant (p<0.05) interaction exist between selenomethionine and omega-3 fatty acid for serum zinc and iron concentration whereas, it was non-significant for serum calcium and copper. Significantly (p<0.05) increased concentration of selenium, zinc, iron and phosphorus was observed in birds fed 0.3 ppm selenomethionine whereas, significantly (p<0.05) increased zinc and iron was observed in birds fed 0.5% omega-3 fatty acid. There was significant (p<0.05) interaction exist between selenomethionine and omega-3 fatty acid for calcium and phosphorus retention percentage. The maximum retention of calcium and phosphorus was recorded in birds supplemented with 0.3 ppm selenomethionine in combination with 0.5% omega-3 fatty acid. There was marked interaction between selenomethionine and omega-3 fatty acid for hemoglobin (Hb), total erythrocytic count, total leukocytic count and platelets (p<0.05) however, it was non-significant for mean corpuscular volume, mean corpuscular Hb, MCH concentration and differential leukocyte count. Dietary selenomethionine significantly (p<0.05) increased the platelet count. Hb concentration was significantly (p<0.05) higher in birds fed 0.5% omega-3 fatty acid whereas, 1% omega-3 fatty acid in the diet drastically increased (p<0.05) the platelet count.

**Conclusion:**

Supplementation of selenomethionine and omega-3 fatty acid improves the nutrient utilization and mineral retention, which subsequently enhance the bone mineralization. Supplementation also helps in combating the stress.

## Introduction

Chicken meat is healthier than other animal protein sources as it contains less fat and cholesterol. It is a source of preformed eicosapentenoic acid and docosahexaenoic acid [1]. Several alternative strategies have been attempted by dietary manipulation to modify the fatty acid composition to produce meat with lower cholesterol and saturated fatty acid. Moreover, the enrichment of poultry meat with omega-3 fatty acid is a novel method to ensure an adequate supply of enriched food products to fulfill the growing and driving consumer demand. Major dietary source of omega-3 fatty acids are fish oil, vegetable oils (principally linseed oil), walnuts, etc. Many studies revealed that chicken meat enriched with eicosapentenoic acid and docosahexenoic acid through addition of fish oil [2] are prone to oxidative destruction and subsequently produce off-tastes and foul odors, thus reducing consumer acceptability. In this regards various strategies have been made to enrich poultry meat with omega-3 fatty acid while maintaining its sensory attributes [3]. Linseed meal is an important ingredient in cattle feed but its use in poultry feed is limited due to the presence of a cyanogenic glucoside, linamarin. It is rich in linolenic acid and converted into eicosapentenoic acid and docosahexenoic acid in the body through various biochemical pathways. Saleh [4] reported that supplementation of n-3 lipid in broilers is profitable, which might be due to better feed conversion ratio.

Along with omega-3 fatty acid the selenium is also an essential trace mineral and has a profound effect on performance and immunity. Selenium and vitamin E deficiency together in poultry causes exudative ­diathesis, pancreatic dystrophy and nutritional muscular dystrophy [5]. Selenium is an integral part of the enzyme known as glutathione peroxidase which are involved in the cell antioxidant defense mechanism and prevent the cells from free radical damage. Therefore, the current experimental trial was conducted to study the effect of selenomethionine and omega-3 fatty on serum mineral profile and nutrient utilization of broiler chicken.

## Materials and Methods

The experiment was conducted in the Department of Animal Nutrition, College of Veterinary Science and Animal Husbandry, Anjora, Durg, Chhattisgarh, India.

### Ethical approval

This research work was carried out as per the ethical guidelines, and the birds were sacrifices as per standard procedure.

### Experimental design and housing

The present study was a 2×3 factorial arrangement of two levels of selenomethionine (0 and 0.3 ppm) and three levels of omega-3 fatty acid (0, 0.5 and 1%). Day-old Vencobb broiler chicks (n=180), were randomly assigned in six treatment groups of 30 chicks in each (three replicates of 10 birds/treatment). The trial lasted for 42 days. Birds were raised under deep liter system. Birds were vaccinated against Ranikhet disease (F strain) and infectious bursal disease on day 7 and day 14, respectively. A booster dose for Ranikhet disease was given on day 28.

### Treatments and additives

Nutrients composition of diets for chicks at 0-42 days old was based on the National Research Council [6] recommendations. Ingredient and proximate composition of broiler’s diet were shown in Tables-1 and 2, respectively. Treatment groups followed of: Group I was control without extra supplementation of either selenomethionine or omega-3 fatty acid. Group II, III, IV, V and VI were supplemented with 0 ppm selenomethionine with 0.5% omega-3 fatty acid, 0 ppm selenomethionine with 1% omega-3 fatty acid, 0.3 ppm selenomethionine with 0% omega-3 fatty acid, 0.3 ppm selenomethionine with 0.5% omega-3 fatty acid and 0.3 ppm selenomethionine with 1% omega-3 fatty acid, respectively. Linseed oil was used as a source of omega-3 fatty acid while sel-plex is used for selenomethionine supplementation.

**Table-1:** Ingredient composition of broiler starter, grower and finisher diet (on % DM basis).

Feed ingredients	Broiler starter	Broiler grower	Broiler finisher
Yellow maize	53	56.40	59.70
Soya DOC	36.40	33.40	26.30
Deoiled rice bran	2.40	-	-
Fish meal	2	2	5
Soyabean oil	2.50	4.60	5.5
Dicalcium phosphate	1.70	1.60	1.30
Limestone powder	0.70	0.70	0.70
DL-methionine	0.28	0.26	0.22
Lysine	0.02	-	0.17
Soda-bi-carb	0.17	0.16	0.23
Salt	0.28	0.33	0.26
Premix*	0.56	0.58	0.63

* Trace mineral premix mg/kg diet: Mg 300, Mn 55, Fe 56, Zn 30, Cu 4; vitamin premix per kg diet: Vitamin A 8250 IU, vitamin K 1 mg, vitamin E 26.84 mg, vitamin B_12_ mg, vitamin B_2_ 4 mg, vitamin B12 100 µg, niacin 60 mg, pantothenic acid 10 mg; choline 500 mg and 30 ppm salinomycin (coxistac 12%), 55 ppm bacitracin methylene di salicyclate (BMD110), Soya DOC=De-oiled cake, DM=Dry matter

**Table-2:** Proximate composition of broiler starter, grower and finisher rations (% on DM basis).

Particulars	Broiler starter	Broiler grower	Broiler finisher
Crude protein	22.90	21.40	19.65
Crude fibre	3.72	3.33	3.25
Ether extract	5.39	7.41	8.48
Total ash	7.32	6.68	6.63
Acid insoluble ash	1.66	1.78	1.39
Nitrogen free extract**	60.67	61.18	61.99
Calcium	0.94	0.85	0.95
Phosphorus	0.75	0.70	0.74
ME (kcal/kg)**	2970	3050	3123

** Calculated value, ME=Metabolisable energy, DM=Dry matter

### Nutrient utilization

A metabolic trial of 3 days was conducted at the end of the experiment (40-42 days). During trial excreta from each group, was collected replicate wise. Daily feed offered and left over were recorded. A representative sample of feed offered and left over was collected daily for laboratory analysis. The excreta were collected once at the end of trial and oven dried at 60°C for the determination of proximate principles and energy contents. Other portions of fresh samples were subjected to analysis for nitrogen [7], calcium [8], and phosphorus [9] content.

### Serum mineral profile

Blood samples were collected at the end of experimental trial from three birds/replicate and serum was separated as per standard procedure. Serum mineral profile was determined by using atomic absorption spectrophotometer (Electronics Corporation of India Ltd., Hyderabad, Model: AAS-4141).

### Hematology

Blood samples were collected from the jugular vein in heparinized vials (heparin @10 IU/ml of blood) for hematological studies. The hematological observations were recorded in three birds randomly selected from each replicate on 42^nd^ day of the experiment. Hemoglobin (Hb), total erythrocytic count (TEC), total leukocytic count (TLC), platelet count, mean corpuscular hemoglobin (MCH), MCH concentration (MCHC) and differential leukocyte count (DLC) were performed as per the method described by Jain [10].

### Statistical analysis

The data obtained were subjected to statistical analysis by the software SPSS version 10 [11] following 2×3 factorial design. Levels of significance were calculated as per the standard method described by Duncan [12] whenever any effect was found significant.

## Results

### Serum mineral profile

The effect of dietary supplementation of selenomethionine and omega-3 fatty acid on serum mineral profile has been presented in Table-3. There was significant (p<0.05) interaction between selenomethionine and omega-3 fatty acid for serum zinc and iron whereas it was non-significant for serum calcium and copper concentration. Significantly (p<0.05) increased level of serum selenium, zinc, iron and phosphorus was observed in birds fed 0.3 ppm selenomethionine in comparison with group in which selenomethionine was not supplemented. Significantly (p<0.05) increased concentration of zinc and iron could also be noticed in birds fed 0.5% omega-3 fatty acid in the diet.

**Table-3:** The effect of selenomethionine and omega-3 fatty acid on serum mineral concentration of broiler chickens.

Minerals	Selenomethionine (0 ppm)			Selenomethionine (0.3 ppm)			Significance		
		
	Omega-3 fatty acid (0%)	Omega-3 fatty acid (0.5%)	Omega-3 fatty acid (1%)	Omega-3 fatty acid (0%)	Omega-3 fatty acid (0.5%)	Omega-3 fatty acid (1%)	Seleno-methionine	Omega-3 fatty acid	Selenomethionine× Omega-3 fatty acid
Se (ppm)	0.17^ay^±0.02	0.18^ay^±0.01	0.18^ay^±0.01	0.32^az^±0.03	0.32^az^±0.03	0.34^az^±0.02	*	NS	NS
Zn (ppm)	0.60^ay^±0.1	0.64^by^±0.09	0.59^ay^±0.13	0.68^az^±0.08	0.72^bz^±0.11	0.72^bz^±0.1	*	*	*
Cu (ppm)	0.16^a^±0.02	0.18^a^±0.01	0.17^a^±0.02	0.18^a^±0.01	0.17^a^±0.02	0.18^a^±0.02	NS	NS	NS
Fe (μg/ml)	34.72^ay^±1.09	37.25^by^±0.98	36.84^by^±1.26	37.58^az^±1.45	40.28^bz^±1.11	39.48^bz^±1.15	*	*	*
Ca (mg/dl)	10.65^a^±0.01	10.66^a^±0.01	10.64^a^±0.02	10.64^a^±0.06	10.71^a^±0.04	10.66^a^±0.05	NS	NS	NS
P (mg/dl)	7.52^ay^±0.37	7.84^az^±0.19	7.76^az^±0.14	7.82^az^±0.1	7.78^ay^±0.14	7.64^ay^±0.18	*	NS	NS

Means within row with different superscripts

abc (Omega-3 fatty acid) and superscript

yz (selenomethionine) differ significantly.

* p<0.05. NS=Non-significance

### Nutrient utilization

The intake, outgo and balance of nitrogen, calcium and phosphorus in chickens have been presented in Table-4. There was significant (p<0.05) interaction exist between selenomethionine and omega-3 fatty acid for calcium and phosphorus retention percentage. The retention percentage of calcium was significant due to selenomethionine alone whereas, it was non-significant for nitrogen and phosphorus. The maximum retention of calcium and phosphorus was recorded in birds supplemented with 0.3 ppm selenomethionine in combination with 0.5% omega-3 fatty acid.

**Table-4:** The effect of selenomethionine and omega-3 fatty acid on nutrient utilization of broiler chickens.

Particulars	Selenomethionine (0 ppm)			Selenomethionine (0.3 ppm)			Significance		
		
	Omega-3 fatty acid (0%)	Omega-3 fatty acid (0.5%)	Omega-3 fatty acid (1%)	Omega-3 fatty acid (0%)	Omega-3 fatty acid (0.5%)	Omega-3 fatty acid (1%)	Seleno-methionine	Omega-3 fatty acid	Selenomethionine× Omega-3 fatty acid
Nitrogen (g/day)									
Intake	3.62^ay^±2.74	4.13^b^±5.13	3.96^b^±3.36	3.80^az^±5.19	4.05^b^±7.41	3.78^a^±4.97	*	*	*
Excreted	0.78^a^±3.38	0.85^a^±3.87	0.82^a^±4.10	0.79^a^±3.83	0.83^a^±5.44	0.78^a^±3.45	NS	NS	NS
Retained	2.84^ay^±4.49	3.28^a^±4.67	3.14^a^±5.61	3.01^az^±7.19	3.22^a^±3.25	3.00^a^±6.33	*	*	NS
Retention (%)	78.45^a^±1.58	79.41^a^±3.65	79.29^a^±3.54	79.21^a^±4.91	79.50^a^±1.13	79.36^a^±3.51	NS	NS	NS
Calcium (g/day)									
Intake	1.05^ay^±1.68	1.28^b^±6.35	1.27^b^±4.81	1.17^az^±7.86	1.28^b^±9.54	1.18^a^±5.78	*	*	*
Excreted	0.62^a^±3.61	0.70^b^±4.02	0.70^b^±4.30	0.65^a^±4.98	0.68^b^±4.38	0.65^a^±4.01	NS	*	*
Retained	0.43^ay^±1.67	0.58^b^±5.65	0.57^b^±7.12	0.52^az^±4.63	0.60^b^±2.71	0.53^a^±3.64	*	*	*
Retention (%)	40.00^ay^±2.06	45.31^b^±3.60	44.88^b^±3.49	44.44^az^±4.29	46.87^b^±1.58	44.91^a^±1.07	*	*	*
Phosphorus (g/day)									
Intake	0.54^a^±7.03	0.62^b^±4.69	0.62^b^±4.64	0.56^a^±8.05	0.61^b^±5.99	0.56^a^±8.66	NS	*	*
Excreted	0.34^a^±5.00	0.37^b^±4.98	0.38^b^±4.47	0.35^a^±5.07	0.35^a^±3.31	0.34^a^±6.08	NS	*	NS
Retained	0.20^a^±5.95	0.25^b^±3.57	0.24^b^±7.22	0.21^a^±5.18	0.26^b^±4.91	0.22^a^±5.07	NS	*	*
Retention (%)	37.03^a^±4.21	40.32^a^±2.06	38.70^a^±6.90	37.5^a^±4.11	42.62^b^±7.58	39.28^a^±3.43	NS	*	*

Means within row with different superscripts

abc (Omega-3 fatty acid) and superscript

yz (selenomethionine) differ significantly.

* p<0.05). NS=Nonsignificance

### Hematological profile

The data pertaining to hematological profile has been presented in Table-5. There was marked interaction between selenomethionine and omega-3 fatty acid for Hb, TEC, TLC and platelets (p<0.05) however it was non-significant for mean corpuscular volume (MCV), MCH, MCHC, and DLC. Dietary selenomethionine alone has no significant effect on Hb, TEC, platelets, MCH, MCHC and DLC, however, TLC and platelets count were significantly (p<0.05) higher due to its supplementation. The Hb concentration was significantly (p<0.05) higher due to 0.5% omega-3 fatty acid whereas, 1% omega-3 fatty acid in the diet drastically increased (p<0.05) the platelets counts. The individual supplementation of omega-3 fatty acid has no significant effect on MCV, MCH, MCHC, and DLC.

**Table-5:** The effect of selenomethionine and omega-3 fatty acid on hematological profile of broiler chickens.

Particulars	Selenomethionine (0 ppm)			Selenomethionine (0.3 ppm)			Significance		
		
	Omega-3 fatty acid (0%)	Omega-3 fatty acid (0.5%)	Omega-3 fatty acid (1%)	Omea-3 fatty acid (0%)	Omega-3 fatty acid (0.5%)	Omega-3 fatty acid (1%)	Seleno-methionine	Omega-3 fatty acid	Selenomethionine× Omega-3 fatty acid
Hb (g/dl)	9.30^a^±0.05	12.40^c^±0.18	11.10^b^±0.09	9.30^a^±0.06	11.7^b^±0.03	11.1^b^±0.06	NS	*	*
TEC (10^6^/mm^3^)	2.13^a^±0.08	2.74^c^±0.03	2.52^b^±0.13	2.21^a^±0.09	2.95^c^±0.04	2.62^b^±0.10	NS	*	*
TLC (10^3^/mm^3^)	134.0^ay^±4.12	147.8^b^±3.29	145.2^b^±2.84	140.8^az^±3.6	148.3^c^±2.97	145.4^b^±3.18	*	*	*
Platelets (10^3^/mm^3^)	38.0^ay^±1.02	52.0^bz^±2.11	66.0^cz^±3.24	61.00^cy^±4.15	44.00^by^±5.19	39.00^ay^±4.47	*	*	*
MCV (mm^3^)	131.0^a^±5.56	136.0^a^±1.69	135.0^a^±2.48	132.0^a^±5.17	133.0^a^±3.44	134.0^a^±1.95	NS	NS	NS
MCH (Pg/dl)	43.6^a^±1.68	45.3^a^±0.34	44.1^a^±1.76	42.2^a^±1.98	43.7^a^±1.04	44.4^a^±0.81	NS	NS	NS
MCHC (g/dl)	33.3^a^±1.08	31.9^a^±2.44	31.4^a^±2.68	32.6^a^±0.55	29.9^a^±2.06	32.3^a^±1.13	NS	NS	NS
DLC (%)									
Lymphocyte	51.25^a^±1.36	52.10^a^±1.14	52.50^a^±0.99	51.70^a^±0.57	52.50^a^±0.33	52.25^a^±0.45	NS	NS	NS
Heterophil	38.50^a^±0.39	38.50^a^±0.31	37.50^a^±0.75	39.00^a^±0.54	38.00^a^±0.60	37.75^a^±0.46	NS	NS	NS
Monocyte	8.0^a^±0.35	7.25^a^±0.45	7.50^a^±0.45	7.25^a^±0.45	7.0^a^±0.40	7.50^a^±0.50	NS	NS	NS
Eosinophil	2.25^a^±0.25	2.15^a^±0.15	2.50^a^±0.50	2.05^a^±0.05	2.50^a^±0.25	2.50^a^±0.30	NS	NS	NS
Basophil	0	0	0	0	0	0	NS	NS	NS
H/L ratio	0.75^a^±0.02	0.73^a^±0.02	0.71^a^±0.03	0.75^a^±0.02	0.72^a^±0.01	0.72^a^±0.02	NS	NS	NS

Means within row with different superscripts

abc (Omega-3 fatty acid) and superscript

yz (selenomethionine) differ significantly.

* p<0.05. NS=Nonsignificance, Hb=Hemoglobin, TEC=Total erythrocytic count, TLC=Total leukocytic count, MCV=Mean corpuscular volume, MCH=Mean corpuscular hemoglobin, MCHC=MCH concentration, DLC=Differential leukocyte count, H/L=Heterophil to lymphocyte

## Discussion

Broiler meat are more prone to oxidative deterioration due to a higher concentration of polyunsaturated fatty acid (PUFA). Among all the PUFA, the omega-3 fatty acid has received considerable attention in the past decade due to reducing the susceptibility toward obesity, coronary artery diseases, hypertension and diabetes [13]. This knowledge has increased the demand for enriched food products through manipulation of both the quantity and quality of lipid composition of chicken meat [14].

There are three important essential omega-3 fatty acids: α-linolenic acid, eicosapentaenoic acid, and docosahexaenoic acid. Human body cannot synthesize omega-3 fatty acids, but synthesize 20-carbon unsaturated omega-3 fatty acids i.e., eicosapentaenoic acid and 22-carbon docosahexaenoic acid from the 18-carbon α-linolenic acid, therefore one way to do this to feed birds with linolenic acid-rich diet [15]. Consumer awareness due to the importance of omega-3 fatty acids toward health benefit has been rising and animal products such as milk, meat and egg can be naturally enriched with omega-3 fatty acids by supplementing the omega-3 fatty acid in their diet.

The present findings regarding serum mineral profile is corroborated with Naylor *et al*. [16] who reported that dietary selenomethionine with and without omega-fatty acid increased the level of selenium in the serum and meat tissue of broilers. Iron, copper, and zinc [17] levels in the serum as well as meat was also found to be higher which might be due to increased bioavailability of this mineral to the birds. Similar findings were reported in the present experiment. Gallardo *et al*. [18] reported that dietary canola oil increased the content of omega-9 and omega-3 fatty acids and decreased the content of omega-6 fatty acids in plasma of broiler chickens. The addition of tuna oil could also be recommendable to increase omega-3 fatty acid in broiler meat to provide a healthier functional food to consumer [19].

Naik *et al*. [20] reported that dietary organic selenium significantly (p<0.01) increased the selenium content of meat.

Dietary selenomethionine and omega-3 fatty acid caused relatively more phosphorus balance when compared with the control group [16], which is in accordance with the current findings. A diet more in unsaturated fat positively affected bone mineralization and consequently structural and mechanical properties of bones [21]. In other studies, the results indicated that dietary lipids, depending upon the type and quantity to be ingest, may enhance or suppress the bone growth and also modulate bone mineralization process [22]. Khare and Baghel [23] concluded that supplementation of selenium in the diet of poultry enhances the growth rate as well as nutrient retention in the broiler birds.

Many researchers have found that heterophil to lymphocyte (H/L) ratio can be a good indicator of stress [24]. During the stress, the number of heterophils per unit of blood increases and lymphocytes decreases in birds but the ratio of these cell types is less variable and thus a better measure than individual cell numbers. Altered H/L ratio have been observed in response to thermal stress. Basophil numbers can also be increased and is associated with acute stress. In the current study, the ratio of H/L was non-significant indicating birds were in the normal physiological status without stress. In fact, the selenomethionine along with omega-3 fatty acid might have an effect to reduce the stress. In contrast with present findings [25] reported that as the level of organic selenium increased in the diet heterophil and H/L ratio significantly (p<0.01) decreased, whereas monocyte and eosinophil count significantly (p<0.01) increased. da Silva *et al*. [26] also reported that broilers receiving organic selenium also had the best H/L ratio.

## Conclusion

The results clearly indicated the effect of selenomethionine and omega-3 fatty acid in broiler chickens. Thus, it may be concluded that selenomethionine and omega-3 fatty acid enriched broilers diet improve serum mineral profile, nutrient utilization and may be beneficial for human health.

## Author’s Contributions

PK carried out the experiment. SPT designed and guided the experiment. TS and SKN helped in the collection and analysis of the data. All authors read and approved the final manuscript.
